# News and Events of the Week

**Published:** 1910-10-22

**Authors:** 


### News and Events of the Week.

The following are the week's arrangements at the Medical Graduates' College and Polyclinic, 22 Chenies Street, W.C. : Monday, October 24, at 4 p.m., Dr. J. H. Sequeira, Clinique (Skin); at 5.15 p.m., Dr. J. S. Risien Russell, "Diagnosis of Disseminated Sclerosis." Tuesday, at 4 p.m., Dr. C. O. Hawthorne, Clinique (Med.); at 5.15 p.m., Dr. Leonard Guthrie, "Tetany and Allied Affections." Wednesday, at 4 p.m., Mr. C. W. Rowntree, Clinique (Surg.); at 5.15 p.m., Mr. Lockhart Mummery, "Diagnosis in Diseases of the Rectum and Anus." Thursday, at 4 p.m., Dr. Clive Rivière, Clinique (Med.); at 5.15 p.m., Dr. Rozenraad (Homburg), "Obesity, and Some Changes in Metabolism." Friday, at 4 p.m., Dr. Dan Mackenzie, Clinique (Ear, Nose, and Throat).

The following are the arrangements for next week at the West London Postgraduate College : Demonstrations by the Surgical Registrar, at 10 a.m., on October 24 and 27; Demonstration by the Medical Registrar, at 10 a.m., on October 28; Demonstration of Minor Operations, at 11.30 a.m., on October 25; Pathological Demonstration at 12 noon, on October 24; and a Lecture by Dr. Grainger Stewart on "Practical Medicine," at 12.15 p.m., on October 26. Lectures will be given at 5 p.m. on October 24, by Dr. Beddard, on "Practical Medicine" (Lecture 2); October 25, by Dr. Low, on "Insects as Carriers of Disease in Tropical Medicine"; October 26, by Mr. Lloyd Williams, on "Methods of Extraction of the Teeth, When and How"; October 27, by Dr. Morton, on "X-Ray Examination of the Digestive System"; October 28, by Dr. Davis, on "Diseases of the Ear, the Lines on which Treatment should be Based."

Dr. William Mackintosh Gilmour, M.D., of Holmcliffe, Gower Drive, Pollokshields, Glasgow, for forty years Government Certifying Factory Surgeon for the districts of Govan and Whiteinch, who died on June 19, aged sixty-eight, has left personal estate in the United Kingdom valued at £22,880.

It is announced that the Territorial Decoration has been conferred upon the following medical officers of the Territorial Force : Transport Officer and Honorary Major W. Gardner, 1st West Riding Field Ambulance; Lieut.-Col. T. H. Openshaw, C.M.G., M.B., F.R.C.S. (attached to the Lincolnshire Yeomanry); Major W. C. Bentley, M.D. (attached to the 6th Battalion Northumberland Fusiliers); Major J. T. Thompson, M.B. (attached to the 5th Battalion Welsh Regiment); Major G. Williamson, M.B. (attached to the 4th Battalion Gordon Highlanders); and Major D. McMillan, M.B. (deceased).

"National Health."—This useful and interesting little monthly is the official organ of the Women's Imperial Health Association, of whose multifarious activities in educating the public in sound hygiene we made mention some weeks ago. The journal is full of instructive reading, and the October issue contains among other interesting articles three of prime importance : one by Mr. Cowley on "Disinfection of Schools in Relation to Public Health," one by an anonymous contributor on "The Defects of Private School Buildings," and another on "Public Swimming Baths." The paper is written throughout in a simple and popular manner and is sold at a penny. It ought to have a very useful function and to become a valuable adjunct to the movement that the Association is promoting.

At the quarterly meeting of the Council of the Royal College of Surgeons, with Mr. Henry T. Butlin in the chair, it was determined to recognise the Great Northern Central Hospital, Holloway, as a place of instruction for the final examination for the Fellowship.

Dr. John Edward Platt, M.D., M.S., F.R.C.S., of High Street, Chorlton-on-Medlock, Manchester, Chairman of the Medical Board of the Manchester Royal Infirmary, lecturer on practical surgery at the University of Manchester, joint editor of the *Manchester Medical Chronicle*, who died on August 3, aged forty-four, has left estate valued at £11,518 gross, and £10,500 net.

The museum of the Royal College of Surgeons has received a collection of sketches of early cancer of the tongue and conditions which may be mistaken for early cancer from the President, from Professor F. W. Bird, of Melbourne, his collection of radiographs of anatomical and pathological specimens, and from Dr. Seligmann fifteen New Guinea skulls. In each case the Council expressed its thanks.

The Page May Memorial Lectures in Physiology will be delivered at University College on the following Mondays and Tuesdays at 4.30 p.m.—October 24 and 25, November 7 and 8, November 28 and 29. The first course, dealing with neurology, will be delivered by Professor C. S. Sherrington, of the University of Liverpool, and will be open to the public without fee. A syllabus can be obtained on application to the Secretary of University College.

Sir Thomas Barlow, Bart., M.D., F.R.S., at a meeting at the Royal College of Physicians, has been elected President of the coming International Congress of Medicine. Mr. G. H. Makins, C.B., has been elected treasurer, and Dr. Herringham secretary. A large organising committee also has been appointed. The date of the seventeenth Congress, which will be held in London during the summer of 1913, was left in the hands of the officers mentioned.

The eleventh French Congress of Medicine was held at Paris from October 13 to 15, under the presidency of Dr. Landouzy, dean of the Paris Faculty of Medicine. Three questions outlined by the preceding Congress were discussed—namely, bradycardia, by Drs. Vaquez and Esmein, of Paris; the treatment of symptomatic epilepsy, by Dr. Sonques, of Paris, and Dr. Vires, of Montpellier; and the liver and spleen in pathology, by Drs. Gilbert and Lereboullet, of Paris, and Dr. Roch, of Geneva. In addition discussions were held on the accidents due to serumtherapy, on acute cerebro-spinal meningitis, on tuberculin therapy, and on the functions of the pancreas. Independently of the discussions on reports and on questions set down in the daily programme, opportunity was given for presenting papers on any medical subject. A French school of stomatology is to be opened in Paris on October 15, with the object of providing instruction for students and practitioners of medicine. To the school will be attached a dispensary for the treatment of diseases of the mouth and teeth. Instruction will be given in clinical and technical methods, in prosthesis, orthodontia, and all the practical work of the speciality.

Pringle's book on "Fractures and their Treatment," which was reviewed in our issue of October 15, is published at 15s. net, not 12s. 6d. as stated in the review.

Mr. Frederick Weatherly, M.R.C.S., of Hillside, Portishead, Somerset, for many years a member of the Somerset County Council, who died on August 11, aged ninety, left estate valued at £7,889 gross, and £6,956 net.

Dr. Henry A. Miers, F.R.S., Principal of the University of London, opened the winter session of the London School of Tropical Medicine, Connaught Road, Albert Dock. Other speakers included Sir West Ridgeway, Lord Sheffield, and Mr. W. H. Lever, the Chairman of the Liverpool School of Tropical Medicine. Mr. Lever advocated a more extensive advertisement of the London and Liverpool schools, on the ground that their want of money was due largely to the wide ignorance which prevailed as to their work.

A number of medical men in general sympathy with the Minority Report of the Poor Law Commission have formed a committee to assist those who are engaged in bringing these proposals favourably before the public. In addition to the successful reception given to the members of the British Medical Association attending the annual meeting in July, several meetings of the Committee have taken place. The Committee will be pleased to arrange for members of one or more of its number to attend any discussions on the Reports of the Poor Law Commission or cognate subjects if desired, and also to open discussions on the Minority Report. Inquiries should be addressed to Mr. Somerville Hastings, F.R.C.S., 51 New Cavendish Street, W., or to Dr. Beckett-Overy, 30 Harley Street, W.

At the recent annual dinner of the Society of Medical Officers of Health, Dr. W. G. Willoughby (the President) occupied the chair, and the company included Sir Dyce and Lady Duckworth, Mrs. W. G. Willoughby, the Rev. Dr. and Mrs. J. M. Willoughby, Sir Lauder Brunton, Mr. F. Hollins (Chairman of the Eastbourne Public Health Committee), Sir Shirley and Lady Murphy, Mr. and Mrs. H. T. Butlin, Mr. B. Broadbent (Huddersfield), the Mayor of Battersea, Dr. Priestley (Hon. Secretary), and Mr. W. A. Lawton (Secretary). Sir Lauder Brunton, responding to the toast of "The Visitors," proposed by the President, said that the National League for Physical Education and Improvement was co-operating with the medical officers of health of the country in an endeavour to prevent microbic infection. If pure milk could be obtained the tale of infant mortality in this country would be greatly diminished.

